# Clinically Manifest Infections Do Not Increase the Relapse Risk in People with Multiple Sclerosis Treated with Disease-Modifying Therapies: A Prospective Study †

**DOI:** 10.3390/jcm12031023

**Published:** 2023-01-28

**Authors:** Giuseppina Miele, Simone Cepparulo, Gianmarco Abbadessa, Luigi Lavorgna, Maddalena Sparaco, Vittorio Simeon, Lorenzo Guizzaro, Simona Bonavita

**Affiliations:** 1 Department of Advanced Medical and Surgical Sciences, University of Campania Luigi Vanvitelli, 80131 Naples, Italy; 2 Neurology Unit, Ospedale del Mare-A.S.L Na1-Centro, 80147 Naples, Italy; 3 Medical Statistics Unit, Department of Physical and Mental Health and Preventive Medicine, University of Campania “Luigi Vanvitelli”, Piazza Luigi Miraglia 2, 80138 Naples, Italy; 4 Human Medicines Division, European Medicines Agency, Domenico Scarlattilaan 6, 1083 HS Amsterdam, The Netherlands

**Keywords:** multiple sclerosis, infections, relapses

## Abstract

Background: Many factors are believed to be positively associated with the incidence of relapses in people with multiple sclerosis (MS), including infections. However, their role is still controversial. We aimed to investigate whether symptomatic infections in people with MS increase the risk of relapse in the short, medium, or long term. Materials and Methods: We enrolled consecutive patients with relapsing MS (RMS) from October to December 2018. From enrolment up to September 2020, an online questionnaire investigating the occurrence of infections was sent via WhatsApp^®^ monthly to the enrolled patients, while in-person visits were performed every six months. When patients complained of symptoms compatible with relapses, they attended an extra in-person visit. Results: We enrolled 155 patients with RMS, and 88.38% of patients were treated with disease-modifying therapies. In the dataset, 126,381 total patient days, 78 relapses, and 1202 infections were recorded over a period of about 2 years. No increased risk of relapse after clinically manifest infections was found in the short-, medium-, or long-term period. No correlation was found between all infections and the number of relapses (*p* = 0.212). The main analyses were repeated considering only those infections that had at least two of the following characteristics: duration of infection ≥ 4 days, body temperature > 37° Celsius, and the use of drugs (antibiotics and/or antivirals), and no significant associations were observed. Conclusions: No associations between infections and relapses were observed, likely suggesting that disease-modifying therapies may protect against the risk of relapse potentially triggered by infections.

## 1. Introduction

Multiple sclerosis (MS) is a chronic neurological disease and the most common cause of non-trauma-related disability in young adults [1]. Most of the patients (80–90%) present with a relapsing–remitting (RR) course.

Many factors are believed to be positively associated with the incidence of relapses, including smoking, pollution, low levels of serum vitamin D [2], non-traumatic stressful life events [3,4], postpartum period, vaccinations, and infectious diseases [5].

Many autoimmune disorders, such as MS, are associated with vitamin D deficiency, and several studies have shown that vitamin D has anti-inflammatory effects by suppressing the innate and adaptive immune systems [6]. In the central nervous system, vitamin D modulates neurotrophic factors and suppresses inflammation by modulating T cell trafficking in the central nervous system, inhibiting Th1 cells and IL-10 production. In this way, vitamin D reduces the number of autoreactive T cells in the central nervous system. In addition, demyelination is reduced through activation of microglia by vitamin D. The relationship between vitamin D levels, supplementation, and disease activity in MS patients treated with different drugs has been examined in several studies, reporting that a 10 nmol/L increase in vitamin D concentration can reduce the risk of relapse by 9% [7], and a 10 nmol/L change in Vitamin D concentration resulted in a 15% reduction in the risk of T2 brain lesions among MS patients [8]. Moreover, vitamin D levels are associated with disease activity in MS patients treated with interferon (IFN)-B, glatiramer acetate, fingolimod, natalizumab, and ocrelizumab [9,10,11,12].

Between the 1990s and the 2000s, several studies highlighted a risk of exacerbations after infections; furthermore, in some of these studies, the authors concluded that infections contributed to an increase in clinical disease activity, without impacting the severity of relapses [13,14,15]. More specifically, Andersen and colleagues reported that relapses are more frequent after viral infections, with a relative relapse risk ratio of 1.3 during a 4-week post-infection period [13].

Despite several studies on this topic, the effect of infections on relapse incidence in people with MS is still controversial [14,15,16,17,18,19,20] due mainly to study limitations such as a small sample size [13,14,15,16,19], the evaluation of limited infections (respiratory or urinary tract infections) [10,13], no investigation of the severity of infectious symptoms, and no correction for seasonality or solar hours (except in the study of Tremlett and colleagues [17]).

Previous studies highlighted a positive correlation between upper respiratory tract infections and monthly relapse rates and a negative one with ambient ultraviolet radiation expressed as solar hours [17]; however, seasonal variation in relapse incidence has been reported only in some studies [21,22].

In this study, we aimed at investigating whether symptomatic infections in an MS outpatient population increase the risk of relapse in the short, medium, or long term, accounting for confounding factors.

## 2. Materials and Methods

This was a cohort study. Consecutive patients with a diagnosis of relapsing MS (RMS) attending the MS outpatient clinic of the University of Campania “Luigi Vanvitelli” (Naples, Italy) from October to December 2018 were offered to take part in the study. Ethical approval was obtained from the ethical committee of the University of Campania “Luigi Vanvitelli” (prot. 593/2018). Signed informed consent was obtained from each patient before enrolment in the study in accordance with the Declaration of Helsinki.

We included patients with MS diagnosis according to the revised McDonald criteria [23], with a relapsing course with or without disability progression. We excluded patients who had relapses in the six months prior to study enrollment and infections that had required pharmacological treatment in the previous 12 months; patients with comorbidities that increase the risk of infections (diabetes, chronic obstructive pulmonary disease, renal insufficiency, alcohol abuse) [24]; and patients with infections confirmed as COVID-19 with a molecular swab. No restrictions for sphincteric scores on the Expanded Disability Status Scale (EDSS) and smoker status were applied.

Patients enrolled from October to December 2018 were followed up with clinical in-person visits every six months and received a monthly online questionnaire via WhatsApp^®^ (see supplementary materials) from enrolment to September 2020.

Baseline characteristics including demographic data (age and sex) and MS history (date of MS onset, age at onset, disease duration, ongoing disease-modifying therapy (DMT), and EDSS at enrolment) were collected.

The online questionnaire prepared in Google Forms (accessed on 1 October 2018-https://www.google.it/?client=safari&channel=iphone_bm) contained items to detect the presence of infection and to identify the occurrence of relapses; in detail, the questionnaire inquired about the presence of an infection and its duration, the presence of fever with body temperature measurement, the type of infectious symptoms (respiratory, gastrointestinal, or urinary), and the use of symptomatic drugs and/or antibiotics and/or antivirals (see supplementary materials). Data were recorded anonymously; at the beginning of the questionnaire patients were asked for their first and last name initials and date of birth.

We considered severe infections those that impaired everyday life [25]. Therefore, the severity of infections was defined by the presence of two of the three following characteristics: duration of infection ≥4 days, body temperature > 37° Celsius, and use of drugs (antibiotics and/or antivirals).

In addition, telephone reporting of relapses and infections was encouraged. In case of relapse suspicion, patients were invited to attend the MS outpatient clinic for an in-person visit; if the relapse was confirmed by two neurologists [26], it was included in the collected data. MS neurologists were not blinded to the information about the infections. Pseudorelapses, which might be due to changes in body temperature [27], were excluded.

We considered as the primary outcome the number of relapses after any infectious disease during the entire study period.

According to clinical practice, MRIs were performed once a year; however, since the onset of T1 gadolinium-enhancing lesions and/or new/enlarging T2 lesions could not be dated, no MRI outcomes were considered.

Since a potential confounder could be the hours of light on each day (as an indicator of seasonality), solar hours, and daylight hours in Naples on each day of the study period, the data were retrieved from the National Oceanic and Atmospheric Administration (NOAA) using a method for sun ephemerides calculations (maptools, R package). Changes in DMT were also collected.

Based on previous studies [16,17,18,19,20], we defined the periods at risk (AR) for relapse occurrence of previous infections as: (i) short-term, high-risk period if a relapse occurred between 14 days before and 43 days after infection; (ii) medium term, high risk if relapse occurred between 44 and 168 days after infection; and (iii) long term, high risk if relapse occurred between 168 and 336 days after infection. “Not at risk” periods included all other days.

### Statistical Methods

We aimed to enroll 155 MS patients. The sample size was calculated based on previous studies [17,20].

The characteristics of the study population are presented using descriptive statistics. Mean or median and (interquartile range (IQR) values were calculated for continuous variables and frequencies for categorical variables.

For the primary analysis, a generalized linear mixed model adjusted for the patient (as a random effect), hours of daylight, and DMT (at the time of relapse) as fixed effects was used to evaluate short-, medium-, and long-term association between all infections and relapses. A simpler model without DMT was also explored. A variable indicating whether a date was subject to restrictions due to the COVID-19 pandemic, which occurred during the observation period, was also used in supplementary analyses as a potential confounder due to its obvious effect on infections and unknown role in relapses. These analyses were also performed by restricting the definition of exposure to severe infections, as previously defined.

To evaluate the association between the number of relapses and the number of infections and between the total number of relapses and infections with body temperature higher than 37 °Celsius, nonparametric Kruskal–Wallis tests were performed. The same analysis was used to evaluate the single association between organ-specific infections (infections with respiratory, gastrointestinal, and urinary symptoms) and relapses. A test of coefficient of correlation (rho-Spearman’s) was calculated for each analysis.

Given the data collection methods, missing data almost exclusively affected the infections. The main analysis was conducted under a missing not at random (MNAR) assumption that no response to the online questionnaire indicates no infection in that month. Sensitivity analyses were conducted under a missing completely at random (MCAR) assumption, considering that the absence of response is completely uninformative regarding the occurrence of infection. A generalized linear mixed model was conducted both adjusting for patient, hours of daylight, and DMTs and without adjusting.

Given a relevant true difference in risk between “AR” and “not AR” days, to determine the power of our study (i.e., in particular our number of relapses) to detect it, a simulation approach was used, using our same dataset for all covariates but simulating the relapse (0/1) column based on real risk increases of 1.5, 1.75, 2, 2.5, and 3. The main scenario to interpret power was prespecified at 2×. For all comparisons, the level of α = 0.05 was assumed, and *p*-values were rounded to four decimal places.

## 3. Results

### 3.1. Population

Out of 209 patients screened for eligibility, 155 patients with RMS signed the informed consent forms, answered the online questionnaire at entry, and were enrolled into the study, of whom 125 had RMS without progression and 30 with progression, and 54 patients did not sign the informed consent and were not enrolled.

The mean age of the participants was 35.4 years, and the median baseline EDSS was 3. The average time from diagnosis to study initiation was 13.25 years, and 113 patients were female (Table 1).

In our study, at the enrollment, 88.38% of patients were treated with DMTs, 41.93% were on first-line DMTs (interferons, glatiramer acetate, teriflunomide, dimethyl fumarate), and 46.45% were on second-line DMTs (natalizumab, ocrelizumab, fingolimod, alemtuzumab) (Table 2). In the study period, 13 patients switched to a second-line DMT.

### 3.2. Clinical Relapses and Infections

**Figure 1:**
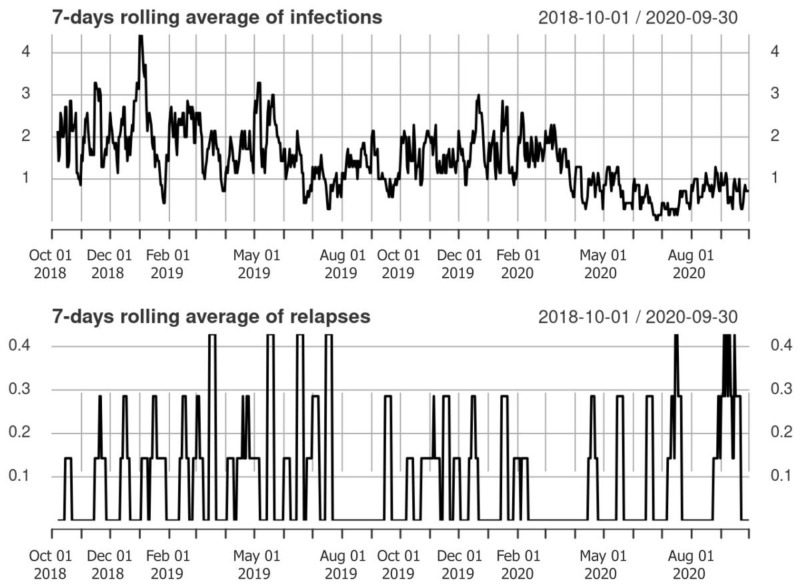
Frequencies of infections and relapses from October 2018 to September 2020.

In the dataset, 126,381 total patient days were registered. The dataset included 78 relapses and 921 infections; frequencies of infections and relapses are shown in Figure 1.

A total of 78 relapses were experienced by 49/155 patients; 27 patients experienced 1 relapse, 15 experienced 2, and 7 experienced 3, averaging 0.50 relapses per patient over the whole study period (range: 0–3). Sensory symptoms were found in 38.46% (30/78) of relapses, motor symptoms in 39.74% (31/78), brainstem or cerebellar symptoms in 15.38% (12/78), and optic neuritis in 6.41% (5/78).

A total of 921 infections were registered, averaging 6 infections per person over the study (range: 0–23). Overall, 17/155 (11%) patients did not report any infections; 138 patients reported a total of 921 episodes of infections including 134 severe cases. The majority of the infections were related to upper respiratory tract symptoms, followed by infections with urinary tract symptoms and gastrointestinal symptoms (Table 3).

### 3.3. Associations between Infections and Relapse Occurrence

**Figure 2:**
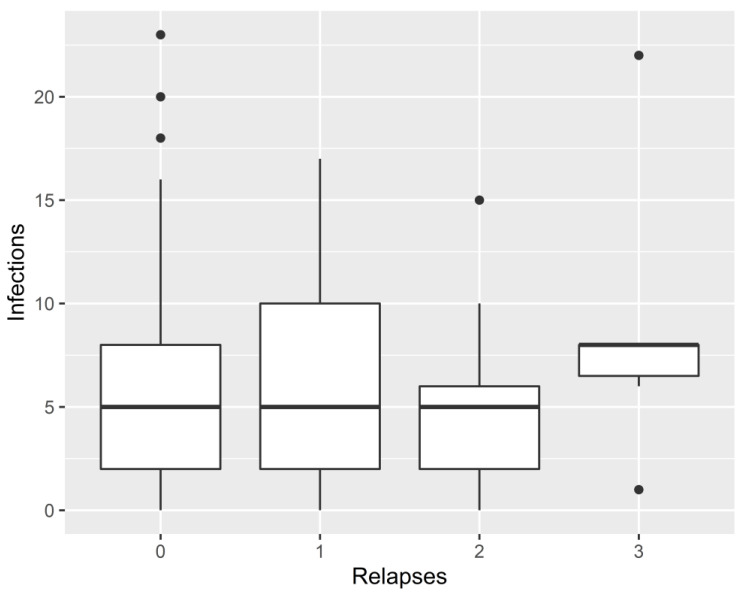
Correlation between the total number of infections and total number of relapses by patients.

The primary analysis, a generalized linear mixed model adjusted for the patient, hours of daylight, and DMTs, did not detect any short-, medium-, or long-term increase in the risk of relapse after clinical infections when analyses were performed using both MNAR and MCAR assumptions. Each analysis was also performed using unadjusted measures, but no correlation was found (Table S1, see supplementary material). No correlation between the total number of infections and the total number of relapses was found (*p* = 0.212) (Figure 2).

**Figure 3:**
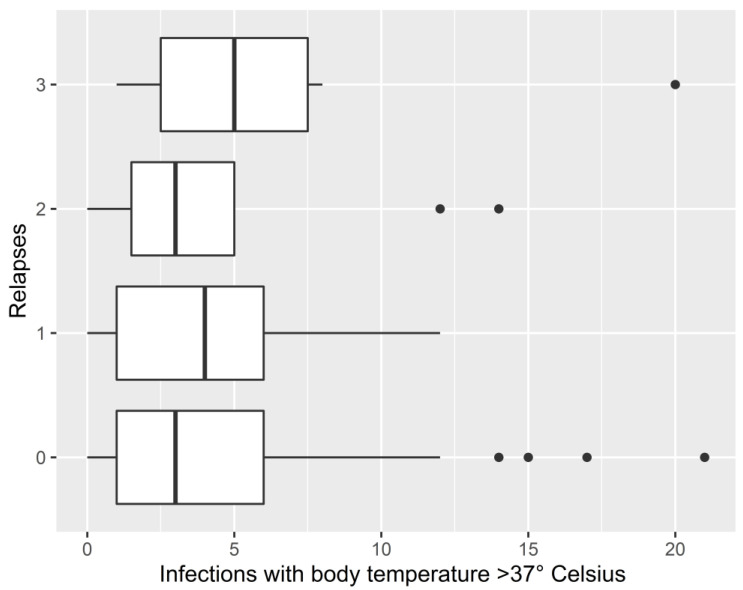
Correlation between the total number of infections with body temperature >37° Celsius and the total number of relapses by patients.

Likewise, no relationship was found between the number of relapses and the number of infections when considering only infections with body temperature >37 ° Celsius (*p* = 0.591) (Figure 3).

Furthermore, no statistically significant relationship was found when analyses were carried out comparing the number of relapses and the type of infection (classified according to infections with respiratory, gastrointestinal, or urinary symptoms; *p* = 0.459, *p* = 0.317, and *p* = 0.215, respectively). The main analyses were repeated considering only severe infections, and no association was observed with the risk of relapse in the short, medium, or long term (Table S2, see supplementary material).

**Figure 4:**
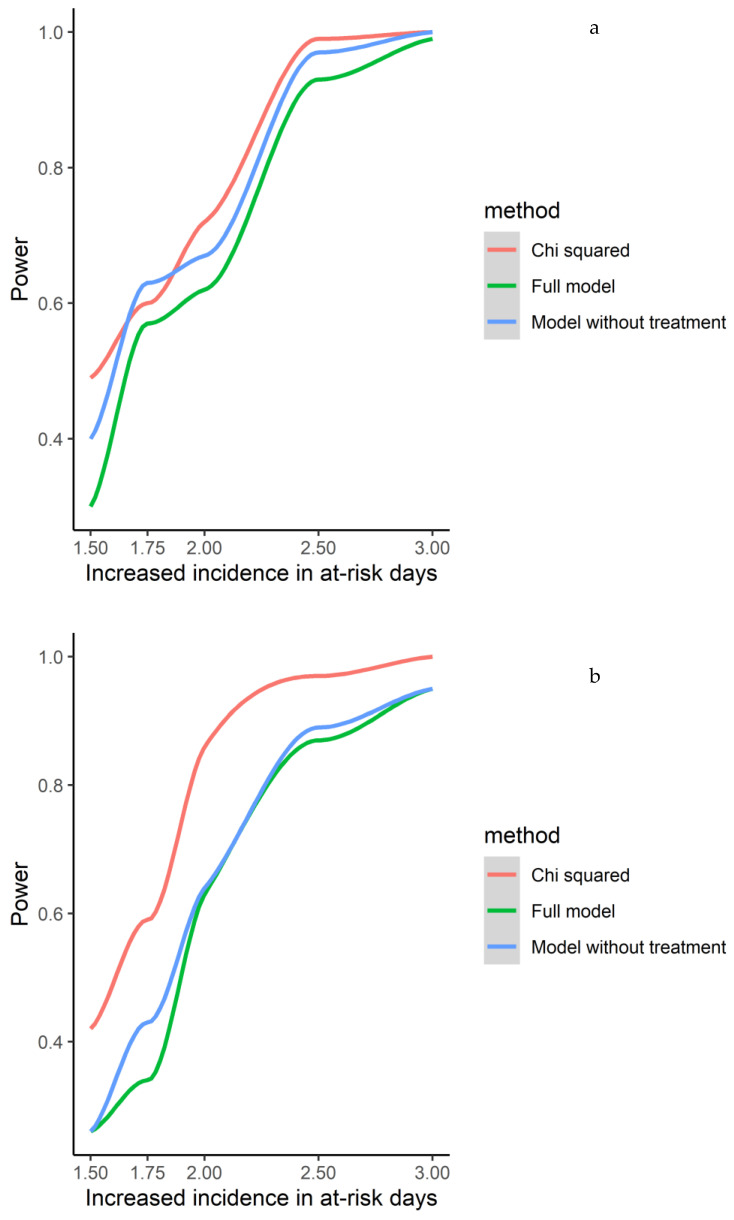

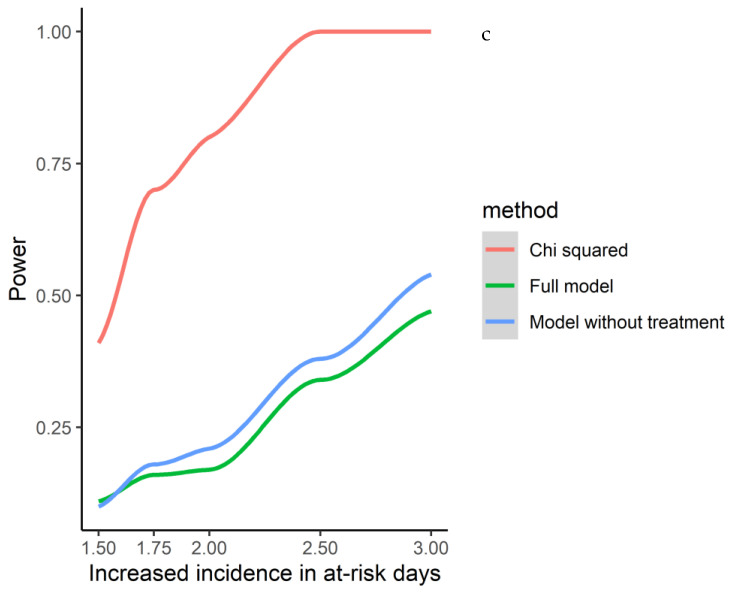
(**a**–**c**) Power analyses were calculated for the full model, for the model with correction of patient and seasonality (but no treatment), and for the basic model (chi-squared analysis).

The power was calculated for the full model, for the model with the correction of the patient and seasonality (but no treatment), and for the basic model (chi-squared analysis). Given that the simulation did not include any real effect of the patient, seasonality, or treatment, a higher power for the basic model was expected when considering the association in the short-, medium-, and long-term (Figure 4).

## 4. Discussion

No association was found between clinical infections and subsequent relapses.

Several lines of evidence have shown a link between the development of an infection and the onset or the exacerbation of autoimmune diseases such as MS [16]. While some viral infections, and in particular some viruses, have been widely recognized to play a role in the onset of MS [28], the role of infections in influencing disease activity in MS is still controversial.

One of the largest studies on this topic was conducted by Sibley et al., which enrolled over 8 years 170 untreated MS patients and 134 healthy controls and showed that annual exacerbation rates were almost threefold in AR periods, defined as two weeks before to five weeks after the onset of a clinical infection, and also that 27% of exacerbations were related to infections [20].

Andersen and colleagues in their prospective study recording upper respiratory and gastrointestinal infections with serological diagnosis in 60 untreated RRMS patients, showed a relative risk of 1.3 to have relapses in the so-called AR period, four weeks after a clinical infection [13].

In the same period, Panitch led a prospective study on 30 RRMS patients, divided into three groups treated with either a high dose of interferon-B 1b (IFN-B lb) (8 MIU), low dose IFN-B lb (1.6 MIU), or placebo and concluded that mild infectious illnesses, largely of nonspecific viral origin, were responsible for most of the exacerbations. He found a strong relationship between MS attacks and upper respiratory tract infections in the AR period (extended from 1 week before the onset of infection to 5 weeks after the onset, with nearly two-thirds of the attacks occurring in AR periods) and pointed out that treatment with a high dose of IFN-B 1B reduced the attack rate [20].

A few years later, Edwards et al. [14] in a prospective study on 41 patients (21 RRMS and 20 secondary progressive MS) randomly assigned to either placebo or 6MIU or 12 MIU of interferon B -1a (IFN-B 1a) treatment, highlighted a 2.1 (*p* = 0.004) relative risk of clinical relapse for upper respiratory tract infections and a higher relative risk of relapse in the AR period (from 14 days before until 14 days after the first symptom) compared with the “not AR” periods (annual attack rates of 5.7 v 1.6, respectively; *p* = 0.006). Their patients were participating in a double-blind placebo-controlled trial of IFN-B 1a, and, at the time of publication, they did not know which patients were on active treatment; for this reason, a subanalysis of treatment was not performed as in Panitch’s study [14,20].

The above-mentioned studies [14,19,20] used different time frames for AR periods because of the difficulties in identifying the exact day of infection onset [14]. More specifically, Edwards and peers by only reducing the frame of the AR period obtained a significant relationship between upper respiratory tract infections and AR period [14], while Andersen et al., using the same time frame as Sibley and colleagues, were unable to reproduce their results [13].

These studies highlighted two fundamental points: (i) all the studies except for that of Sibley et al. had a small sample size and, in some cases, included heterogeneous populations by clinical phenotype, and (ii) the AR periods had different time frames.

In any case, all these studies, although conducted in different ways, point towards a positive correlation between mild infections, in most cases of viral origin, and relapses. These studies were conducted in the 1990s, mainly on untreated patients. Panitch was the first author to observe in the cohort of his treated patients that the number of relapses was lower compared with untreated patients, regardless of the number of infections.

Nearly 80-85% of people with multiple sclerosis experience a relapsing course, and if left untreated, they develop disability over time [29]. Over the past three decades, the treatment of MS has evolved with the development of new disease-modifying therapies targeting various mechanisms and radically changing patient outcomes. Twenty-three DMTs (glatiramer acetate, interferon beta 1a and 1b, natalizumab, cladribine, fingolimod, teriflunomide, alemtuzumab, dimethyl fumarate, ocrelizumab, ozanimod, and ofatumumab) were compared with each other or with a placebo with mixed comparisons for ARR in a recent meta-analysis. This meta-analysis revealed that the risk of relapse for all DMTs was significantly lower than for the placebo, with the exception of Betaseron 50 μg [30].

At the start of our study, 88.38% of patients were treated with DMTs, whereas after one year 92.90% of patients were on DMTs. This could be one of the reasons why we did not observe any correlation, as the effect of DMTs is currently so strong that external factors such as mild or moderate infections do not influence disease activity.

Given that, our study has adequate power to detect strong associations (Figure 4); we trust these results that corroborate the absence of an increased risk of relapse after clinical infections.

Given the correlation between solar hours and infectious risk and relapse, we adjusted results for solar hours to add strength to our analysis (Table S1, see supplementary material).

Unlike our study, Tremlett et al. [17], considering other factors such as serum vitamin D levels and prior erythemal ultraviolet radiation (to evaluate exposition to solar hours), found a positive association between relapse rates and upper respiratory tract infections in a cohort of 199 MS patients (142 with RRMS); however, they found that the strength of association between upper respiratory tract infections and relapses was weaker after adjusting for monthly erythemal ultraviolet radiation. Furthermore, in this study, 78.16% of patients were on DMTs, mainly injectable therapies (INF or glatiramer acetate).

Of note, we have to underline that this cohort of patients is extremely different from ours, as in our cohort almost all patients were treated with DMTs: 88.38 % at the enrollment and 92.90% one year after the study started, with a stronger impact on disease activity, possibly overcoming the detrimental effect of infection in triggering relapses.

One of the major limitations of our study is the modality of the infection recording. This was performed through the administration of an online questionnaire that was sent monthly to the RMS patients of our MS Center. Subsequently, they were not asked to confirm infection by more in-depth investigations.

Unfortunately, vitamin D levels were not available for all the patients, representing a further limitation.

Moreover, this was a single-country, single-center study that may limit generalizability to the MS patient population.

In addition, there were missing data (about 11%) as patients received the questionnaire monthly over a long period (two years). However, the presence of missing data was managed through statistical analysis. The analyses were conducted both considering that the absence of response meant the absence of infection (MNAR) and not attributing any value to the absence of response (MCAR). If the probability of being missing is the same for all cases, then the data are said to be missing completely at random (MCAR), while MNAR means that the probability of being missing varies for some reason and we assume that the reason is the absence of infection. Moreover, this was a monocentric study, and patients with underlying conditions increasing the risk of infections were excluded, and the results might not generalize to that subgroup or in other contexts.

We recognize that these types of studies may suffer from variations in MS severity, infection type, and severity and that pooling all the cases (with all type of variations) and performing the correlation analysis may lead to suboptimal sensitivity for investigating the correlation between infection and relapse. Studying subtypes of infections or patients with different severities of MS, might have improved the statistical power for the correlation, but this would require a larger sample size to be explored. Considering these aspects, we tried to overcome this limitation through the statistical model by adjusting for the patient as a random effect and for sunlight and treatment as fixed effects.

## 5. Conclusions

In conclusion, in our cohort of MS patients, most of them taking DMTs (He or ME DMTs), mild or severe infections were not associated with an increased risk of relapse. Although associations between mild infections and relapse rate in the AR period had been described in the past, we speculate that, currently, the use of effective DMTs may protect against the risk of relapse potentially triggered by infections. Future studies are needed to confirm this hypothesis.

## Figures and Tables

**Table 1:** Demographic characteristics of the whole population.

		Whole Population RMS (N = 155)
Age (years)	Mean (IQR)	35.406 (37.5–49.3)
Female	N (percentage)	113 (72.9%)
EDSS ^1^	Median (IQR)	3 (2–4.5)
Disease duration (years)	Mean (IQR)	13.25 (6.36–20.4)
Patient days reported in the dataset	N	126,381
Total number of relapses reported in the dataset	N	78
Total number of infections reported in the dataset	N	1202

^1^ EDSS, Expanded Disability Status Scale.

**Table 2:** Patient distribution according to ongoing DMTs at study enrollment and after one year.

Number of Patients on DMTs ^1^ at the Enrollment (2018) N (Percentage)				Number of Patients on DMTs ^1^ after One Year from the Enrollment N (Percentage)			
No DMTs	18/155 (11.61%)	No DMTs	18/155 (11.61%)	No DMTs	11/155 (7.10%)	No DMTs	11/155 (7.10%)
Interferon	28/155 (18.06%)	I Line Therapies	65/155 (41.93%)	Interferon	24 (15.48%)	I Line Therapies	59/155 (38.06%)
Glatiramer Acetate	19/155 (12.26%)			Glatiramer Acetate	17 (10.97%)		
Teriflunimide	12/155 (7.74%)			Teriflunimide	12 (7.74%)		
Dimethyl-fumarate	6/155 (3.87%)			Dimethyl-fumarate	6 (3.87%)		
Fingolimod	48/155 (30.97%)	II Line Therapies	72/155 (46.45%)	Fingolimod	52 (33.55%)	II Line Therapies	85/155 (54.83%)
Natalizumab	11/155 (7.10%)			Natalizumab	10 (6.45%)		
Ocrelizumab	4/155 (2.58%)			Ocrelizumab	22 (14.2%)		
Alemtuzumab	2/155 (1.29%)			Alemtuzumab	1/155 (0.65%)		
Others (Rituximab)	7/155 (4.52%)			Others (Rituximab)	0/155 (0)		

^1^ DMTs, disease-modifying therapies.

**Table 3:** The proportion of type and severity of infections according to symptom severity.

Type of Infections		
Infections with upper respiratory tract symptoms	N (percentage)	741/921 (80.4%)
Infections with urinary tract symptoms	N (percentage)	402/921 (43.6%)
Infections with gastrointestinal symptoms	N (percentage)	360/921 (39.1%)
Characteristics of infections		
Duration longer than 4 days	N (percentage)	482/921 (52.33%)
Body temperature >37° Celsius	N (percentage)	82/921 (8.9%)
The use of antibiotics and/or antivirals	N (percentage)	157/921 (17%)
Severe infections ^1^	N (percentage)	134/921 (14.54%)

^1^ Severe infections, defined by the presence of two of the three following characteristics: duration longer than 4 days; body temperature > 37° Celsius, and the use of antibiotics and/or antivirals.

## Data Availability

Not applicable.

## Supplementary Materials

The following supporting information can be downloaded at: https://www.mdpi.com/article/10.3390/jcm12031023/s1, Table S1: Generalized linear mixed model adjusted for patient, hours of daylight, and DMTs to detect short-, medium-, or long-term increase in the risk of relapse after clinically manifest infections; Table S2: Generalized linear mixed model adjusted for patient, hours of daylight, and DMTs to detect short-, medium-, or long-term increase in the risk of relapse after clinically manifest infections, considering only infections that had at least two of these three characteristics: duration of infection ≥4 days, body temperature > 37° Celsius, and use of antibiotics or antivirals; Online Questionnaire.
