# Supplementary material for: Clinically Manifest Infections Do Not Increase the Relapse Risk in People with Multiple Sclerosis Treated with Disease-Modifying Therapies: A Prospective Study
Source: J Clin Med. 2023 Jan 28;12(3):1023. doi: 10.3390/jcm12031023 (PMC9918238; doi:10.3390/jcm12031023)
Supplement: Supplementary file 1 [file jcm-12-01023-s001.zip › jcm-2118039-supplementary.pdf]

Supplementary materials

Table S1. Generalized linear mixed model adjusted for patient, hours of daylight and DMTs, to detect short- mid- or long-term increase in risk of relapses after clinically manifest infections.

	Estimate	SE	z-value	p
Short-term association<br><br>(43 days after and 14 days before the onset of an infection)<br><br>MNAR analysis	0.157	0.295	0.532	0.595
Short-term association<br><br>(43 days after and 14 days before the onset of an infection)<br><br>MCAR analysis	0.439	0.464	0.946	0.344
Mid-term association<br><br>(168 and 43 days after the onset of an infection)<br><br>MNAR analysis	0.222	0.354	0.627	0.531
Mid-term association<br><br>(168 and 43 days after the onset of an infection)<br><br>MCAR analysis	8.142^{e-01}	9.273^{e-01}	0.878	0.380
Long-term association<br><br>(336 and 168 days after the onset of an infection)<br><br>MNAR analysis	9.847^{e-01}	6.457^{e-01}	1.525	0.127
Long-term association<br><br>(336 and 168 days after the onset of an infection)<br><br>MCAR analysis	3.660^{e+01}	1.447^{e+06}	0.000	1.000

MNAR, missing not at random; MCAR, missing completely at random; SE, standard error.

Table S2. Generalized linear mixed model adjusted for patient, hours of daylight and DMTs, to detect short- mid- or long-term increase in risk of relapses after clinically manifest infections, considering only infections that had at least two of these three characteristics: duration of infection ≥4 days, body temperature > 37° Celsius and use antibiotics or antivirals..

	Estimate	SE	z-value	p
Short-term association<br><br>(43 days after and 14 days before the onset of an infection)<br><br>*MNAR analysis	-0.157	0.295	-0.532	0.595
Mid-term association<br><br>(168 and 43 days after the onset of an infection)<br><br>*MNAR analysis	-0.222	0.354	-0.627	0.531
Long-term association<br><br>(336 and 168 days after the onset of an infection)<br><br>*MNAR analysis	-9.847^{e-01}	6.457^{e-01}	-1.525	0.127

MNAR, missing not at random; SE, standard error.

Online Questionnaire

- Inserisca le iniziali di nome e cognome e data di nascita

1. *Relativamente al mese appena trascorso, ricorda di aver avuto infezioni riguardanti l'apparato respiratorio (anche un banale raffreddore, tosse, faringite), gastroenterico (vomito, diarrea con o senza febbre) o genitourinario (bruciore urinario, prurito)?*
   Si
   No (se la risposta è "NO", bisogna barrare la casella e concludere il questionario cliccando "invia" al termine della pagina)

2. *Segnali la data di inizio dei sintomi*

3. *Segnali la data del termine dei sintomi*

4. *Se durante l'episodio infettivo ha avuto anche febbre, segnali la temperatura massima raggiunta*
   Non ho avuto febbre
   Ho avuto la febbre, la temperatura massima è stata di 37°C
   Ho avuto la febbre, la temperatura massima è stata di 38°C
   Ho avuto la febbre, la temperatura massima è stata di 39°C
   Ho avuto la febbre, la temperatura massima è stata maggiore di 39°C

5. *Ha avuto sintomi riguardanti l'APPARATO RESPIRATORIO? Scelga i sintomi con attenzione può barrare più caselle*
   NON HO AVUTO SINTOMI RIGUARDANTI QUESTO APPARATO
   Sensazione di naso chiuso
   Presenza di muchi nasali fluidi/acquosi
   Muchi nasali densi (giallo-verdastri)
   Naso che cola
   Mal di gola
   Muchi in gola
   Placche alla gola
   Dolore alle tonsille
   Dolore alla deglutizione
   Voce rauca
   Sensazione di "ossa rotte"
   Tosse secca senza muchi o con muchi fluidi
   Tosse grassa, catarrale, con muchi densi

6. *Ha avuto sintomi riguardanti l'APPARATO GASTRO-INTESTINALE? Scelga i sintomi con attenzione (può barrare più caselle)*
   NON HO AVUTO SINTOMI RIGUARDANTI QUESTO APPARATO
   Dolore addominale
   Nausea
   Vomito
   Diarrea

7. *Ha avuto sintomi riguardanti l'APPARATO GENITO-URINARIO? scelga i sintomi con attenzione (può barrare più caselle)*
   NON HO AVUTO SINTOMI RIGUARDANTI QUESTO APPARATO
   Prurito intimo
   Esigenza di urinare spesso
   Bruciore urinario

Sangue nelle urine

8. *Ha assunto farmaci (antibiotici, cortisone o altro)? se "SI" specifichi nome del farmaco, posologia e durata del trattamento. Se non ha assunto farmaci scriva "NO"*

9. *Il suo medico le ha fatto diagnosi di INFLUENZA o ALTRO?*

10. *Si sono modificati i sintomi della Sclerosi Multipla durante l'episodio infettivo?*
    SI, si sono aggravati sintomi precedentemente già presenti
    NO, non si sono aggravati i sintomi preesistenti e non ho avuto ricadute
    Ho avuto una ricaduta (in questo caso, se non lo ha già fatto, segnali l'accaduto allo staff medico)

11. *Il suo contributo per noi è importante. Se ha avuto anche altri episodi infettivi oltre a quello appena segnalato, dovrà cliccare "submit" o "invia" e poi "invia un'altra risposta". Nello spazio che segue può annotare, consigliare o precisare... GRAZIE DELLA COLLABORAZIONE!*
